# Supplementary material for: Electric field assisted alignment of monoatomic carbon chains
Source: Sci Rep. 2020 Jun 16;10:9709. doi: 10.1038/s41598-020-65356-8 (PMC7297712; doi:10.1038/s41598-020-65356-8)
Supplement: Supplementary file 1 — Supplementary Information. [file 41598_2020_65356_MOESM1_ESM.pdf]

Electric field assisted alignment of monoatomic carbon chains

Stella Kutrovskaya,* Igor Chestnov,† Anton Osipov, Vlad Samyshkin, Irina Sapegina, Alexey Kavokin, and Alexey Kucherik

Here we describe the approach that allowed us to collect the data of the orientation of deposited carbyne - gold nanoparticles (NPs) complexes by TEM scaling. Figure 1 illustrates the sputtering set-up geometry (a) with reference points marking the electric field orientation on the surface of the sample. (b) shows the sample fixed on the TEM holder as well at the frame chosen for the orientation measurements, (c) shows an example of the TEM image of the deposited nanostructures with the greed line showing the orientation of a specific structure, red line showing the electric field orientation. Figure 2 shows the magnified TEM image of an individual deposited nanostructure composed by a carbyne thread connecting

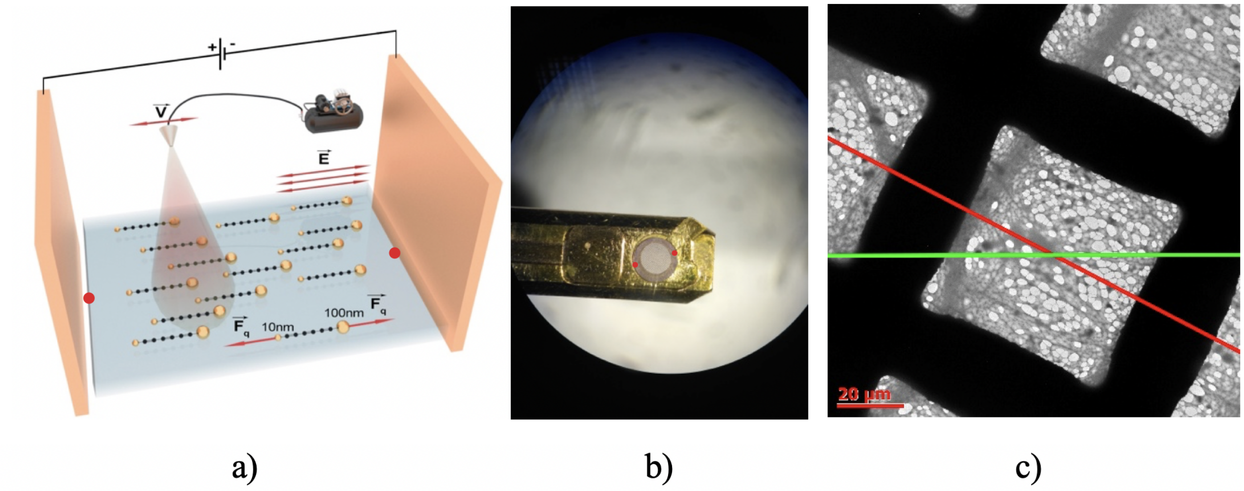

FIG. 1. The method of measurement of the angle of orientation of the deposited nanodipoles with respect to the electric field orientation during the sputtering process. (a) The scheme of the sputtering setup showing the reference points that mark the direction corresponding to the electric field orientation of the surface of the substrate. (b) The view of a sample fixed on a TEM holder, where two red points set up the reference frame. (c) The green line shows the orientation of one of the deposited nanostructures, while the red line shows the orientation of the electric field. The angle between red and green lines is measured for each of studied nanostructures in order to obtain the statistical distribution.

two gold NPs of different sizes. The green line connects the centers of NPs. The inset shows the angle between the green line and the red line that corresponds to the electric field orientation. Figure 3 shows TEM images of a reference sample where carbyne-gold nanostructures were deposited on a substrate in the absence of external electric field. One can clearly see that no any alignment or orientation of deposited structures is observed in this case.

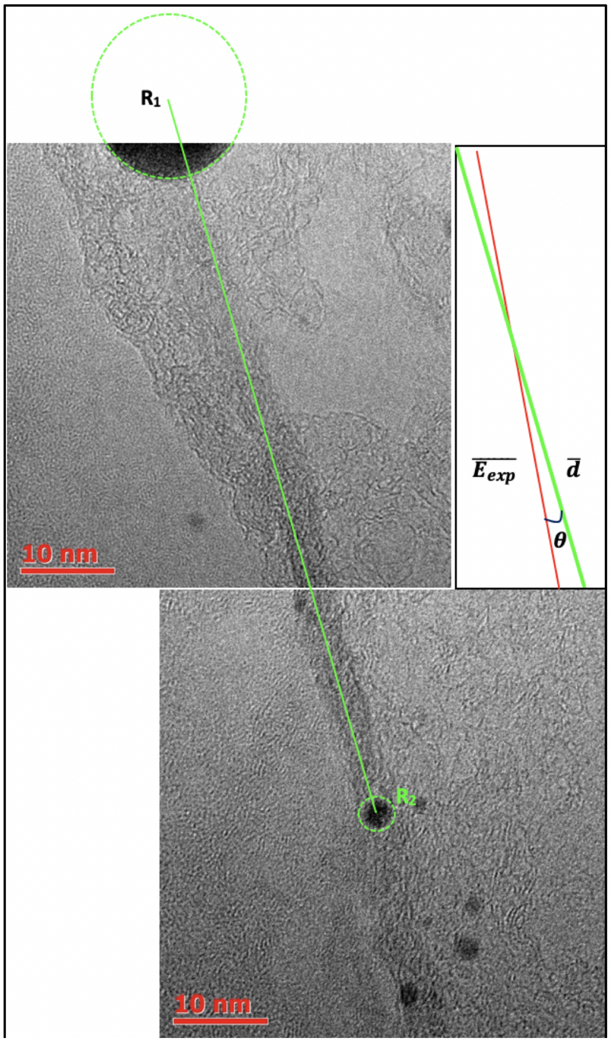

FIG. 2. The angle θ between the orientation of the deposited carbyne-gold nanostructure and the electric field is extracted from the analyses of high-resolution TEM images. The green line connecting the centers of NPs on the TEM image is superposed to the red line showing the electric field orientation in the inset. The values of θ angles characterising the orientation of all studied nanostructures are stored in the computer memory and used to produce the experimental distribution function.

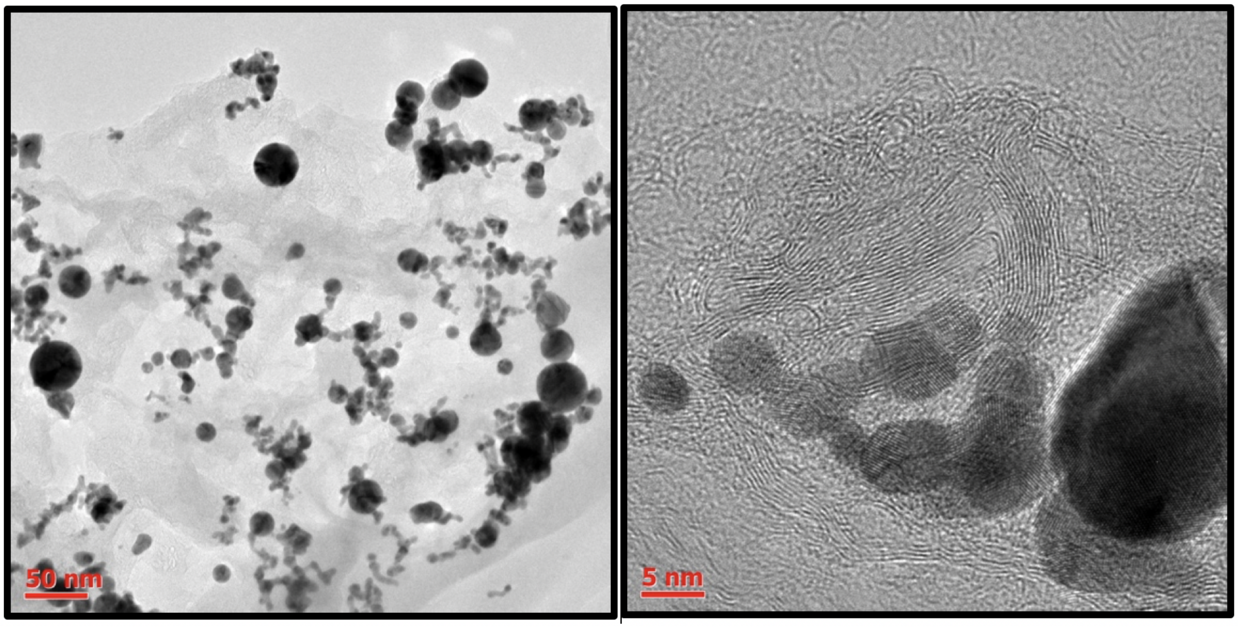

FIG. 3. Experimental results on the reference sample where the deposition of the monoatomic carbon chains end-capped with Au NPs has been done by sputtering in the absence of the static electric field. Left and right panels show lower and higher spatial resolution images, respectively.
